# A survey for potentially zoonotic gastrointestinal parasites in domestic cavies in Cameroon (Central Africa)

**DOI:** 10.1186/s12917-017-1096-2

**Published:** 2017-06-26

**Authors:** Felix Meutchieye, Marc K. Kouam, Emile Miegoué, Terence T. Nguafack, Joseph Tchoumboué, Alexis Téguia, Georgios Théodoropoulos

**Affiliations:** 1 Department of Animal Production, Faculty of Agronomy and Agricultural Sciences, PO BOX 188, Dschang, Cameroon; 2 Center for Research on Filariases and other Tropical Diseases (CRFilMT), P.O. Box 5797, Yaoundé, Cameroon; 3 Department of Anatomy and Physiology of Farm Animals, Faculty of Animal Science and Aquaculture, Agricultural University of Athens, 75 Iera Odos St, Votanikos, 11855 Athens, Greece

**Keywords:** Zoonoses, Domestic cavies, *Giardia*/*Cryptosporidium*, Cameroon

## Abstract

**Background:**

Farm animals are usually suspected to transmit infections to humans. Domestic cavies (*Cavia porcellus*) are hosts to a variety of pathogens, some of which are zoonotic. Several parasites including the protozoa *Giardia* spp. and *Cryptosporidium* spp. may be causative agents of gastrointestinal disorders in domestic cavies and humans. The aim of the study was to investigate the occurrence of potentially zoonotic protozoa as well as any potential zoonotic gastrointestinal parasite in domestic cavies raised under a semi extensive system in the rural areas of Cameroon.

**Results:**

*Giardia*/*Cryptosporidium* antigens were detected in 12.90% of cavies. Helminthe eggs were found in 1.52% of animals. The prevalence of *Paraspidodera uncinata*, *Heligmosomoides polygyrus* (also known as *Nematospiroides dubius*) and *Trichuris sp.* was 1% (4/397), 0.3% (1/397), and 0.3% (1/397), respectively. Presence of *Giardia*/*Cryptosporidium* was unrelated to the occurrence of diarrhea, as none of the positive samples was from a diarrheic individual.

**Conclusion:**

Domestic cavies are hosts of *Giardia*/*Cryptosporidium* and appear as potential source of human giardiasis, cryptosporidiosis and infection with *H. polygyrus* in Cameroon. In keeping with the One Health Initiative, veterinarians and medical doctors should collaborate to address the problem of *Giardia* and *Cryptosporidium* infection in cavies and cavy breeders both in Cameroon and other countries with a similar cavy breeding system. Follow-up studies are required to further taxonomically characterize these cavy parasites and to determine their routes of transmission to humans.

**Electronic supplementary material:**

The online version of this article (doi:10.1186/s12917-017-1096-2) contains supplementary material, which is available to authorized users.

## Background

Zoonotic hazards have always been a concern to public health officials, animal feed industries, food animal industries and consumers all over the world. The One Heath Initiative, which is a strategy to minimize the global impact of zoonotic and infectious diseases at the animal-human-ecosystem interface through interdisciplinary collaboration, is a response to this shared concern. *Cryptosporidium* spp. and *Giardia* spp. are intestinal protozoa parasites recognized as prevalent and wide-spread enteropathogens of humans and many species of mammals [1–3]. They are commonly identified in domestic livestock, where they shed large numbers of oocysts and cysts. Contamination of water and food with livestock feces containing *Cryptosporidium* oocysts and *Giardia* cysts could occur via routes that span the entire food production continuum [4, 5]. Outbreaks of water and food borne diseases caused by *Cryptosporidium* spp. and *Giardia* spp. are well documented [6], but the role of domestic cavies (*Cavia porcellus*) commonly known as guinea pigs in the maintenance and transmission of these parasites is poorly reported. Nowadays domestic cavies are no longer limited to a small-scale production for research or game purposes only but have become an important item in the livestock production sector in many countries [7–9]. In Cameroon, the population of cavies is increasing, for instance from 30, 293 to 35, 256 heads in 2011 and 2012 respectively [10], following the decision of the government to increase the source of income and meat in rural areas through mini-livestock species husbandry such as cavies [11]. With the projected increase in cavy production in the country, there is a concern about the emergence of zoonotic infection especially giardiosis and cryptosporidiosis which have previously been reported in many settings in domestic cavies [12–16]. Furthermore, complaints from farmers on diarrheal stools in both children and cavies during past surveys reinforced the suspicion of the occurrence of these parasites on cavy farms, since *Giardia* spp. and *Cryptosporidium* spp. are known to cause diarrhea in children [2, 17, 18] and cavies [12, 13]. In addition, the environmental burden of *Cryptosporidium* oocysts and *Giardia* cysts from fecal waste reported in Cameroon [19–21] urged us to study whether *Cryptosporidium* and *Giardia* occur in cavy husbandry in Cameroon, in order to raise awareness of their potential threat on public health and on cavy industry under development. Thus, the main objective of this study was to detect any presence of *Giardia*, *Cryptosporidium* or any potential zoonotic gastrointestinal parasite in domestic cavies raised by farmers in the rural areas of the country.

## Methods

### Study area and farms

The study was carried out between June and July 2014 in the western highlands of Cameroon, an agro-ecological zone covering the North West and West administrative regions of the country, located between 5°20′-7° north latitude and 9°40′-11°10′ east longitude. The North West and West regions are the largest cavy production zone of the country, and account for 94.1% of the national production in 2012 [10]. The region is characterized by a high relief, and the climate is of the Sudano-Guinean type. There is one rainy season, from mid March to Mid November and one dry season from mid November to mid March. Humidity varies from 80 to 98%. Annual precipitation ranges from 1500 to 2500 mm while minimum and maximum temperature are 10 °C and 34 °C respectively [22]. The human population is estimated at 1.82 million inhabitants, and is one of the highest population densities in the country, with at least 79 inhabitants per km2 and a population growth rate of 3.1% [23].

The study was conducted in privately-owned farms in rural areas of Menoua and Bamboutos border Divisions. Farms are small holder units also keeping rabbits, sheep, goats, or local fowl breeds. The housing system is either the cage system or in most cases, the free kitchen roaming system; in this latter system, cavies share the kitchen floor with the local fowl and/or small ruminants, and feed on kitchen wastes, forages and rarely concentrate. The husbandry system is essentially semi-extensive.

### Study design and sample collection

There is no central registry of farms in Cameroon so private farms were located and visited using a snowball sampling technique whereby a livestock farmer, when located helped to locate the next farm and so on. Fecal samples were randomly collected regardless of any endogenous or exogenous factors such as breed, age, gender, or housing system in a cross sectional survey from village households who owned cavies and wished to participate in the study. Cavies were randomly caught and individually put into a large and clean bucket to defecate. Soon after defecation, individual fecal material from non-diarrhoeic and diarrhoeic cavies was carefully collected into capped, plastic sample containers containing 10% formalin, then sealed. The sample size of interest was the one to detect a disease in a population. Therefore, the sample size was determined based on the formula for sample size calculation [24] as follows: n = (1-(1-p)^1/d^)* (N- (d-1)/2) where n = sample size, p = probability of finding at least one case (95%), d = number of cases in the population, and N the population size. The cavy population size in the West Region was obtained from the Cameroon National Institute of Statistics [10] as 19,948 individuals. A previous study [25] in the area showed an overall prevalence of gastrointestinal parasites to be 12.9%; thus the estimated number of cases (d) in the population was determined as 2574. Finally, the computed sample size was determined as 22. However, in order to get some idea of the proportion of infection in the population, the sample size was increased depending on the economical means, to 397 for the parasitological test, and 93 for the serological test.

### *Cryptosporidium* spp. and *Giardia* spp. antigen detection

Fecal samples were tested for the presence of specific *Giardia* and *Cryptosporidium* specific antigens using a commercial coproantigen ELISA test kit (Chek® *Giardia/Cryptosporidium* Microplate Assay, Wampole®), according to the manufacturer’s instructions. The *Giardia*/*Cryptosporidium* Chek® is a solid phase immunoassay for the simultaneous detection of *Giardia* cyst and *Cryptosporidium* oocyst specific antigen in aqueous extracts of fecal specimens. The test uses monoclonal and polyclonal antibodies to cell-surface antigens of *Giardia* cyst and an oocyst antigen of *Cryptosporidium*. The optical density values were obtained using an automatic plate reader (Multiscan RC V1.5.0).

### Detection of gastrointestinal parasite eggs/cysts/oocysts in fecal samples

Fecal samples were analyzed qualitatively using the saturated salt solution (NaCl) as flotation fluid. The simple flotation method was used to detect the parasite eggs, cysts and oocysts which were identified microscopically based on morphology and size [26, 27]. Heavy eggs were screened using the simple sedimentation test, as described by Zajac and Conroy [27]. Slides were mounted and examined at 100 and 400 magnifications.

## Results

In total, 397 fecal samples were collected, of which 93 were analyzed for antigen detection. Of these 93 samples 12 (12.90%) were positive. Since the assay simultaneously detect *Giardia* and *Cryptosporidium* antigen, these positive samples could be only *Giardia* or only *Cryptosporidium* or both *Giardia* and *Cryptosporidium* antigens. All the *Giardia*/*Cryptosporidium* positive samples were from non-diarrheic individuals.

All the samples collected were analyzed for the presence of eggs, cysts and oocysts of gastrointestinal parasites. Helminthe eggs were detected in 6 (1.52%) samples. The helminthes found were *Paraspidodera uncinata* (1%), *Heligmosomoides polygyrus* (Syn. *N. dubius*) (0.3%) and *Trichuris* sp. (0.3%) (Table 1). Cysts of *Giardia*, oocysts of *Cryptosporidium* or eggs of tremadodes were not observed in feces.

**Table 1:** Helminthe infections in domestic cavies in the western highland of Cameroon

Helminthes	Total number of animals examined	Number positive	Percentage (%)	95%CI^a^
*Paraspidodera uncinata*	397	4	1	(0.3–2.7)
*Heligmosomoides polygyrus*	397	1	0.3	(0.0–1.6)
*Trichuris* sp	397	1	0.3	(0.0–1.6)

^a^CI: Confidence interval

## Discussion

There was a need to screen the cavy population for the presence of either *Giardia* or *Cryptosporidium*, and any other gastrointestinal, potential zoonotic parasite, given the importance of cavies in small scale farming in the country.

Positive cases of *Giardia/Cryptosporidium* infection were found, indicating that either *Giardia* or *Cryptosporidium* or both parasites occur in cavy husbandry in the country.

Among zoonotic parasites, the unicellular flagellate *G. intestinalis* (syn. *Giardia duodenalis*, *Giardia lamblia*, *Lamblia lamblia*) is known to cause gastroenteritis in its hosts. Molecular and phylogenetic analyses of *G. duodenalis* isolates identified eight distinct genetic groups (known as assemblages A–H), which differ in their host distribution [28, 29]. Assemblages A and B are the zoonotic assemblages [30] associated with human and domestic animal infections [15]. As Assemblage B has been isolated in domestic cavies [15], the coproantigen Elisa positive result indicates that the domestic cavies could potentially play a role in the zoonotic transmission of giardiasis in the region surveyed. Since giardiasis causes the infected cavies to become weak, moribund or dead [31], there is a possible threat to cavy industry in the country due to the economic harm that might be incurred following animal death and presence of disease. This is supported by complaints from farmers of fever, diarrhea and sudden death in cavies during past surveys [25]. To unveil the true status of giardiasis in domestic cavies in the country, follow-up studies are required to determine the species and the molecular isolate of the *Giardia* parasite present in cavies and human.

The zoonotic transmission of *Cryptosporidium* parasites between farm animals and humans is well documented [3, 5], with *C. parvum* regarded as the most important species occurring between humans and animals [5]. Previous studies to compare *C. parvum* from human and *C. wrairi* from domestic cavies showed very striking similarities and also differences on their infectivity in mice [14, 32]. Though a previous study to resolve the controversy in the taxonomy of *Cryptosporidium* species showed that *C. wrairi* was not a species on its own but one of the genotypes of *C. parvum* [33], *C. wrairi* is now recognized as a separate species [34]. Whether *C. wrairi* is a genotype of the zoonotic *C. parvum* or a species on its own, it is a potential zoonotic protozoa since it has been documented in human and also in cattle [35]. Therefore, a zoonotic transmission of *Cryptosporidum* between human and domestic cavies is possible. Concerning cavy husbandry, coproantigen Elisa showed positive results, indicating that this parasite may be present. Since cryptosporidiasis in domestic cavies cause depression, anorexia, diarrhea, watery caecal contents, and death [12], there is a possibility of economic losses among cavy farmers. Again, this is supported by previous observations in cavy health during our previous survey [25]. As for *Giardia*, no conclusions could be drawn for *Cryptosporidium* until further studies are carried out to show that the genotypes from human and cavies isolates in the area are similar.

The test used for the detection of *Cryptosporidium* and *Giardi*a (oo) cysts was coproantigen ELISA which detect the oo (cyst) antigen, rather than the (oo) cyst itself. Unlike serological tests which detect the antibodies to the pathogen, this test detects a parasite element, indicating a current infection. This test has the advantage of being more sensitive and simple in the detection of *Cryptosporidium* and *Giardia* than microscopy techniques. A negative result for *Cryptosporidium* and *Giardia* with the fecal flotation may be related to the flotation solution (Sodium Chloride) which is likely to distort the (oo) cysts, thus leading to a poor diagnosis of these parasites [27].

The fact that *Giardia/Cryptosporidium* positive samples were all from non-diarrheic individuals suggest that diarrheic stools in cavies might be due to other causes (bacteria, viruses, other parasites, feedstuff and others).

Three helminthe species were detected in the present study: *P. uncinata*, *Heligmosomoides polygyrus* (Syn. *Nematospiroides dubius*) and *Trichuris* sp. *P. uncinata* is a common parasite of domestic cavies which was reported in previous studies [25, 36], indicating that this parasite could be endemic in the area.

*H. pygyrus* (Syn. *N. dubius*) is not known to occur in cavies but is documented as a zoonotic parasite of rats and mice in North America and Europe [37]. Due to the common presence of mice and cavies in the household, the parasite is likely to be spurious in cavies. Nevertheless, since rats, mice and cavies are rodents, cavies stand as potential host for this zoonotic parasite. Until the adult worm is identified in domestic cavies, it will be risky to conclude on a parasite-host relationship between *H. polygyrus* (Syn. *N. dubius*) and cavies. The prevalence was quite low (0.3%) suggesting that the transmission risk is low.

The genus *Trichuris* has been identified in wild cavies (*Cavia apera aperea*) in Peru [38] and in domestic cavies [39]. This genus occurs both in humans and animals but as this parasite was not identified to species level in this work, the zoonotic characteristic of the parasite cannot be discussed. In addition, the infection level was quite low (0.3%) suggesting that the threat to human and cavy husbandry in the study area is also low.

## Conclusion

Domestic cavies are a potential source of human giardiasis, cryptosporidiosis and infection with *H. polygyrus* (Syn. *N. dubius*) in the western highlands of Cameroon. The presence of *Giardia* and/or *Cryptosporidium* in cavies kept in the same living area with humans suggests that transmission of *Giardia* and *Cryptosporidium* between cavies and human is possible. Therefore, good hygiene practices are recommended during and after handling cavies or exposure to their feces. Similarly, living with cavies in the same household or living close to the keeping area as is the case with cavy farmers in the rural areas, should be avoided. These preventive measures are useful not only to control the parasites described in this study, but many other zoonotic agents occurring in cavies. Implementation of these measures will both protect cavy industry and public health. In keeping with the One Health Initiative, veterinarians and medical doctors should collaborate to address the problem of *Giardia* and *Cryptosporidium* infection in cavies and cavy keepers in Cameroon and other countries with a similar cavy production system. Follow-up studies are required to further taxonomically characterize these cavy parasites, and to determine their potential route of transmission to human.

## Additional file

Additional file 1: Values in the table are the optical densities obtained from the analysis of fecal samples for the presence of *Giardia/Cryptosporidium* coproantigen. Values A1 and B1 refer to positive control while value H12 refers to negative control. (XLS 20 kb)

## Abbreviations

BecA: Biosciences Eastern and Central Africa
CSIRO: Commonwealth Scientific and Industrial Research Organization - Australia
DFAT: Department of Foreign Affairs and Trade - Australia
ILRI: International Livestock Research Institute
NaCl: Sodium chloride
